# Silicate Cements*A report of Dr. Vogt’s paper read before The Ontario Dental Society, Toronto, May, 1918, and printed in *Oral Health* for June, 1918.

**Published:** 1918-07

**Authors:** Thomas Cowling


### SILICATE CEMENTS *

THOMAS COWLING, D.D.S.

The study of the chemical and physical properties of the silicates involves a thorough understanding of the chemistry of the oxyphosphates. These are best reviewed under two heads, viz., the powder and the liquid.

The earliest attempts at producing a dental cement consisted of a powder—zinc oxide, and a liquid—phosphoric acid. This acid was in a more or less concentrated form. Experience in the use of these early cements soon showed that there was required, in addition to the zinc oxide, some other material in order that they might be worked with greater ease, and also result in a greater

* A report of Dr. Vogt's paper read before The Ontario Dental Society, Toronto, May, 1918, and printed in *Oral Health* for June, 1918.

degree of permanency. This gave rise to the search for suitable modifiers—a search which has led up to the present-day organizations devoted to this special investigation. As a result of the interest taken in this subject, various substances have been introduced as modifiers. Among these may be mentioned magnesium oxide, bismuth oxide, etc. It was found that when bismuth was used with zinc oxide, that it tended to increase the density and imperviousness of the cement. It was also learned that magnesium had a marked effect upon the time required for the setting of the cement.

The study of the liquid content of the cement had for its object the introduction of modifiers in order to reduce the acidity of the liquid as well as to regulate the rate of setting of the cement. As a result of long and careful research there has been added to the phosphoric acid liquid, certain metallic oxides or hydroxides, such as those of calcium, strontium, zinc, aluminum and berrylium.

It is not easy to explain fully the reaction that takes place when the powder and liquid are mixed together. It may, however, be stated in general terms that the basic zinc oxide reacts with the acid liquid, producing zinc phosphate and water. Such an action is comparable to that which takes place when milk of magnesia (magnesium hydroxide) is used as a mouth wash in order to correct the condition of acid mouth. Here the neutralization of the free acid is brought about with the formation of magnesium salts and water. When we mix a dental cement, the zinc phosphate formed, combines with the water of the liquid to form crystalline hydrated zinc phosphate. [It is important that we observe that all cement liquids contain more or less water, hence if we leave the stoppers out of the liquid bottle there will be evaporation of the water content, and this will result in the weakening of the cement. It is also possible that the liquid may absorb moisture from the atmosphere when conditions are favorable. This is equally detrimental to the strength of the cement.]

The oxyphosphate of zinc cement was found to be too opaque and dead in appearance to warrant its use for esthetic restorations, and an insistent demand was made for a translucent tooth-like filling material which could be inserted in a plastic state. It was also desired that the cement have a natural tooth-like appearance, combined with permanency of form. Because zinc oxide used by itself was not translucent, search had to be made for other materials to meet the requirements of the times, hence the introduction of silicate cements.

Naturally enough, having had experience with the various forms of zinc cements, it seemed reasonable that if a translucent basic substance could be combined with it, which, because of its basic properties, would react with an acidic liquid, then all difficulties would have been solved. Attention was first directed to the metallic oxides and hydroxides, but with the exception of calcium and aluminum, they were found undesirable as chief constituents—calcium oxide (quicklime) because of its caustic properties, and aluminum oxide because of its inactivity. Both of these oxides have also an opaqueness about equal to that of zinc oxide. It was found, however, that aluminum possessed some properties of such a desirable nature that its use was indicated, provided the unattractive qualities could be modified. Hence it was suggested that a combination of these two oxides, calcium and aluminum, be used.

The desired cement was one which would have the durability, appearance and attractiveness of porcelain, so the investigating chemists took up the study of this material, as it alone seemed to offer a possible solution of the problem in hand. Porcelain is made by fusing together silica, kaolin and feldspar. Now kaolin and feldspar are complex aluminum silicates containing more or less potash, aluminum being next to silica in percentage. It was thought then, that the desired dental cement might be secured by combining, if possible, calcium with the porcelain constituents in such a way that they would react with the liquid. Prolonged study of this problem resulted favorably, for it was found that such a reaction was possible, provided a strongly basic melt was made, that is, one in which the proportion of basic oxides or their equivalents were higher than that of silica, an acidic oxide.

As with the zinc oxides, so with the silicates; their introduction was soon followed by the use of modifiers, as a result the present-day silicates may be said to include the oxides of calcium, aluminum, berrylium, strontium, sodium and potassium as basic factors, whilst the oxides of silicon, boron, phosphorus and titanium along with fluorides and fluosilicates furnish the acid characteristics. It must be clearly borne in mind that whatever modifications have been introduced, still, the strongly basic aluminum silicate is always the foundation of the silicate cement, and may be considered as bearing the same fundamental relation to the silicates as zinc oxide does to the oxyphosphates.

When preparing the silicate powder, the raw materials are sifted and mixed, then fused at a temperature of from 2,000 degrees F. to 2,500 degrees F. to a homogenous liquid, thus insuring density and uniformity. This forms the ''melt'' referred to previously. It resembles porcelain in appearance, and is poured from the crucible. The frit is broken up and ground in ball mills, and after sifting, is ready for use.

The liquid differs from that of the oxyphosphates only in its being, in general, a less concentrated solution of acid phosphate and phosphoric acid. An intimate study of the chemistry of the silicates shows that when the liquid and powder are mixed together, the phosphoric acid reacts with the powder, producing the acid phosphates of calcium and aluminum. These acid phosphates and those already present in the liquid react with more powder, resulting in the normal phosphates of calcium and aluminum and water. During these reactions, some free silicic acid separates out, due to the decomposition of the complex silicates by the acid. To the phosphates of calcium and aluminum are due the cementing and adhesive properties of the cement, hence the more complete the reaction between powder and liquid the better is the filling that is produced.

The reaction which takes place when mixing the powder and liquid of a cement is a chemical one. Now, we know that any chemical reaction is dependent upon the number of molecules of the reacting substance which can come into intimate contact with each other. The more thoroughly the powder and liquid are mixed together, the greater is the possibility of fresh molecules of the liquid reaching the new surfaces of the powder.

Temperature also is an important factor in the mixing of cements. In general, a rise in temperature increases the rate of the reaction, therefore it is an advantage to carry out any given operation at as definite a temperature as possible. For this reason, the chilling of the cement mixing slabs is advantageous in the warm weather.

Again, the quantities of powder added to the liquid, the rate at which the powder is added, and the duration of spatulation; all exert an important influence upon the final result. The addition to the liquid of large instead of small portions of powder, tends to produce great heat (heat of neutralization), and the mass does not retain its plasticity for as long a time as it otherwise would. Both the strength and durability of the cement are depreciated by the addition of the powder either too rapidly or too freely. It is best to determine experimentally all these factors and carry out each operation uniformly.

When a silicate cement filling has been inserted in a cavity it may be said to undergo two distinct periods of hardening, viz., the primary setting, which requires from five to twenty minutes, and the secondary hardening, which continues over a longer period—months or even years elapsing before the completion of the final stage. The major part of the hardening, however, is complete in twenty-four hours.

An explanation of these two phases of the hardening process may be offered: The primary stage is analogous to that of the oxyphosphates, and consists in the combination of the metallic phosphates with water; the secondary hardening is probably due to the slow hydration of calcium aluminum silicate and the drying out of colloidal silicic acid.

The repeated references to the importance of water in the hardening process of silicate cements might be interpreted as discounting the use of the rubber dam whilst inserting the filling. Because of this possibility, emphasis must be placed upon the fact that whilst in a plastic condition, silicates are *not* hydraulic, and must be protected from excessive moisture. The reason is that in the mixing and hardening processes the *soluble* acid phosphates are changed into insoluble hydrated phosphates.

On account of the length of time taken for the initial hardening it is best not to attempt the polishing of a silicate filling until it has been in place at least twenty-four hours. A protection of some form of varnish, applied to the rough filling, will aid in carrying it over until such time as it can be properly polished. Engineering practice with Portland cement has shown clearly that it is best to allow the mix to remain undisturbed for some time after the setting process has begun. It is a fair inference then, that this is good practice also with other cements, for the chemistry is the same for all.—*Oral Health.*

